# A distinctive case of giant mastoid osteoma in a young adolescent female

**DOI:** 10.6026/973206300214246

**Published:** 2025-11-15

**Authors:** Gaurav Gupta, Ruchika Agrawal, Sangeeta Gupta, Dharmendra Kumar Pipal, Tanushree Trivedi1, Aditya Singh

**Affiliations:** 1 Department of General Surgery, All India Institute of Medical Sciences (AIIMS) Gorakhpur, Uttar Pradesh, India; 2 Department of ENT, All India Institute of Medical Sciences (AIIMS) Gorakhpur, Uttar Pradesh, India; 3 Department of Physiology, All India Institute of Medical Sciences (AIIMS) Gorakhpur, Uttar Pradesh, India

**Keywords:** Osteoma, mastoid, temporal bone, tumour, giant, post-auricular

## Abstract

Mastoid osteoma is a benign osteoblastic tumour with a low incidence and has rarely been documented in childhood and adolescence. It manifests as a solitary lesion and the size rarely exceeds 3 cm at the time of presentation. The diagnosis is based on clinical and radiological findings. Complete excision of the tumour is the recommended treatment, based on the cosmetic concerns. We report a case of a giant mastoid osteoma in an 11-year-old female and outline its presentation, diagnosis and management.

## Background:

Osteomas are benign, osteoblastic mesenchymal tumours arising from periosteum [1, 2]. Most common locations of osteomas are the skull and sinuses, particularly the frontal sinus [2]. Temporal bone osteomas are rare, accounting for 0.1-1% of all benign tumours of the skull [3]. Moreover, the external auditory canal (EAC) is the predominant site for them in the temporal bone [4, 5]. Extra-canalicular location involving mastoid or the squamous part of the temporal bone is rarer and infrequently reported [6]. Mastoid osteomas usually present as slow-growing, hard, and painless postauricular swelling. These are typically asymptomatic, though cosmetic issues, in more superficial locations, such as a mass in post-auricular area pushing the pinna forward and infections from obstruction of sinuses have been reported [3]. Pain, conductive hearing loss, chronic external otitis, and ear canal obstruction are other functional symptoms reported to be associated with the condition [6]. Computed tomography (CT) scan is the gold standard for diagnosis [7]. Surgical excision is the treatment of choice [8]. The pathophysiology of osteomas remains debatable. Some authors consider osteomas to be congenital lesions, arising from an embryonal cartilaginous rest or a persistent embryological periosteum [9]. Colonic diseases such as Gardner's syndrome and others are linked to osteomas, which may indicate a hereditary component [10]. On the other hand, susceptibility to trauma has also been suggested to contribute to the development of these tumours [11]. The age distribution of osteomas has been reported to be quite variable, with a peak incidence between 30 and 50 years of age [12]. There is a dearth of cases reported at a very young age, as revealed by the literature search [10]. Also, owing to the small size, slow growth and absence of pain, osteomas have usually been described as a fortuitous finding in clinical settings. These solid nodular lesions are usually < 10 mm in diameter. However, large osteomas, though rare, are usually reported owing to the cosmetic concerns. Lesions larger than 30 mm in diameter are considered giant tumours [13]. This case report, to the very best of our knowledge, is the first to describe a case of mastoid osteoma in an 11-year-old female with a large post-auricular protruding mass with maximal diameter exceeding 7 cm. Therefore, it is of interest to report describes a case of giant mastoid osteoma incorporating the clinical presentation, diagnostic process, differential diagnosis and the surgical management done.

## Case report:

An 11-year-old female presented to the surgery outpatient department (OPD) with the chief complaint of left post-auricular swelling, for 2 years. Swelling was painless, gradually progressive, yet the left ear pinna was remarkably pushed forward as a result of the growing mass. There was no history of trauma, otorrhea, dizziness, vomiting, facial weakness, or similar swellings elsewhere in her body. She had no other complaints other than the cosmetic concern. On examination, swelling was bony hard, about 8 x 7 cm in size, painless, immobile with smooth surface and without skin involvement. Both the tympanic membranes were intact, and huge, firm, painless, mass-like lesion was found on the left post-auricular area. Brainstem auditory evoked response was performed prior to the surgery (Figure 1a - see PDF). Latency-intensity curve depicted parallel displacement of the curve plotted for left ear. The degree of deafness was determined by the proportion of the displacement beyond the normal range, which revealed very mild-mild conductive deafness (Figure 1b - see PDF) [14]. Non-contrast computed tomography (NCCT) scan of the head and face with 3-D reconstruction showed a large (7 x 6 cm), well-circumscribed, lobulated hyperdense post-auricular lesion arising from the outer table of mastoid on left side. The diagnosis of a giant mastoid osteoma was determined based on the clinical and radiographic findings. Because of the large size and primarily for cosmetic reasons, she was counselled for surgical excision of the lesion. Excision of tumour was performed under general anaesthesia using post-auricular approach and the bony swelling was dissected from all the sides. The bony lesion was removed from its attachment to the skull using periosteum elevator, chisel and hammer. Mastoid drill was also used to facilitate the removal of osteoma and to prevent opening up of the mastoid. After removal, left over bony spikes were levelled-up with the help of drill. The excised osteoma (6.8 x 6.4 cm in size) was sent for histopathologic examination. Procedure was uneventful, however incision was extended on both the ends as the osteoma was significantly large. Postoperative oedema was present for 7 days which subsided on its own. Sutures were removed on post-operative day 8. The one-month postoperative examination was uneventful. Regular follow-ups are scheduled to detect any recurrences.

## Discussion:

Osteomas are benign tumours of the head and neck, commonly found in the frontal and ethmoidal sinuses, with an exceedingly rare occurrence in the mastoid region [7]. Most commonly reported locations in the temporal bone are external auditory meatus, middle ear, internal auditory canal, styloid process, temporomandibular joint, apex of the petrous temporal bone [3]. It has higher incidence in female patients, predominantly in the 2nd and 3rd decades of life and it is rare in puberty [10]. Our case of mastoid osteoma in an 11-year-old female child, hence, is an even more uncommon presentation. Furthermore, the size of the tumour at the time of presentation albeit quite variable, is usually smaller than 3 cm [15, 16]. The present case presented with a huge mastoid osteoma as the maximal diameter exceeded 7 cm without any pain or other symptoms. Giant osteomas at this location have sparsely been reported [17]. Osteomas of the craniofacial region are usually asymptomatic. Nonetheless, symptoms including facial palsy, ear fullness, pressure-related pain, hearing loss from meatal blockage, and persistent discharge have been reported in few studies [18]. Clinical examination, characteristic histological features and specific radiological findings (with comparatively clearer radiological borders) differentiate mastoid osteoma from other similar presentations including osteoblastic metastasis, osteosarcoma, Paget's disease, ossifying fibroma, osteoid osteoma and giant cell tumour [8]. Etiology of osteoma is not clearly known and is reported to be multifactorial [7]. Congenital origin has been explained by some, while infectious theory, supported by association with recurrent suppurative otitis media has also been reported. Traumatic theory has been based on the cases with subperiosteal bleeding from trauma [9]. Since the patient in our study had no history of infection or trauma, congenital origin was considered the most likely explanation. Syndromic association in the form of Gardner's syndrome has been defined when it is presents along with familial adenomatous polyposis and multiple cutaneous epidermoid cysts [9]. Solitary osteoma with no other supportive findings excluded Gardener's syndrome in this patient. Excellent results have been reported by complete excision of the tumour [8]. The postoperative course has been uneventful for this patient as yet. Mastoid osteomas have also not been associated with malignant transformation in the literatures so far [10].

## Conclusion:

Mastoid osteoma though a slow growing, benign tumour arising from temporal bone, can present with a huge size at a younger age. Clinical and radiological examinations particularly computer tomography renders diagnosis easier. Surgical excision is performed taking into consideration the cosmetic deformity and associated symptoms. Complete excision is associated with good prognosis, rare recurrence and good cosmetic results.
